# Polyethylene Glycol Impacts Conformation and Dynamics of *Escherichia coli* Prolyl-tRNA Synthetase Via Crowding and Confinement Effects

**DOI:** 10.1021/acs.biochem.3c00719

**Published:** 2024-04-12

**Authors:** Jessica Liebau, Bethany F. Laatsch, Joshua Rusnak, Keegan Gunderson, Brianna Finke, Kassandra Bargender, Alex Narkiewicz-Jodko, Katelyn Weeks, Murphi T. Williams, Irina Shulgina, Karin Musier-Forsyth, Sudeep Bhattacharyya, Sanchita Hati

**Affiliations:** † Department of Chemistry and Biochemistry, University of Wisconsin-Eau Claire, Eau Claire, Wisconsin 54702, United States; ‡ Department of Chemistry and Biochemistry and Center for RNA Biology, The Ohio State University, Columbus, Ohio 43210, United States

## Abstract

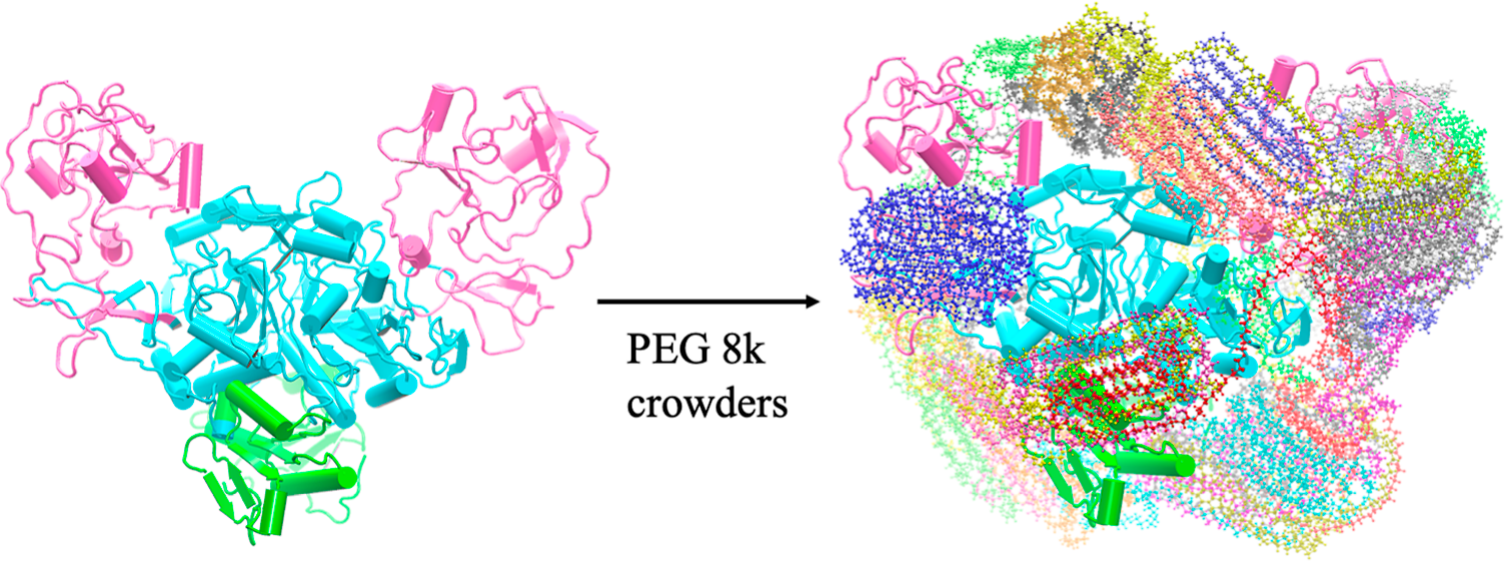

Polyethylene glycol (PEG) is a flexible, nontoxic polymer commonly used in biological and medical research, and it is generally regarded as biologically inert. PEG molecules of variable sizes are also used as crowding agents to mimic intracellular environments. A recent study with PEG crowders revealed decreased catalytic activity of *Escherichia coli* prolyl-tRNA synthetase (Ec ProRS), where the smaller molecular weight PEGs had the maximum impact. The molecular mechanism of the crowding effects of PEGs is not clearly understood. PEG may impact protein conformation and dynamics, thus its function. In the present study, the effects of PEG molecules of various molecular weights and concentrations on the conformation and dynamics of Ec ProRS were investigated using a combined experimental and computational approach including intrinsic tryptophan fluorescence spectroscopy, atomic force microscopy, and atomistic molecular dynamic simulations. Results of the present study suggest that lower molecular weight PEGs in the dilute regime have modest effects on the conformational dynamics of Ec ProRS but impact the catalytic function primarily via the excluded volume effect; they form large clusters blocking the active site pocket. In contrast, the larger molecular weight PEGs in dilute to semidilute regimes have a significant impact on the protein’s conformational dynamics; they wrap on the protein surface through noncovalent interactions. Thus, lower-molecular-weight PEG molecules impact protein dynamics and function via crowding effects, whereas larger PEGs induce confinement effects. These results have implications for the development of inhibitors for protein targets in a crowded cellular environment.

## Introduction

Enzymes are not rigid and static; rather, they are intrinsically flexible and dynamic in nature. Enzyme dynamics on variable time scales are encoded in the primary structure of a protein and translated to its three-dimensional folds. These intrinsic dynamics are known to impact catalytic function.^1−3^ The interplay of structure, dynamics, and function has been widely studied via experimental and computational approaches, predominantly under dilute conditions. However, the correlation among structure, dynamics, and function is expected to vary between the in vitro dilute conditions and the in vivo crowded conditions containing proteins, lipids, nucleic acids, and other biomolecules^4,5^ to concentrations of 100–450 g/L.^6,7^ It is proposed by Agarwal^3^ that networks of residues are involved in providing thermodynamical coupling between the catalytic pocket of an enzyme and the hydration shell and bulk solvent surrounding it. The fluctuations in solvent molecules and cosolutes, and the mode of their interactions with an enzyme could influence its conformational dynamics and thereby its catalytic function.^3^ In recent years, an increased number of computational and experimental studies have focused on understanding the impact of molecular crowding on the interplay among enzyme structure, dynamics, and function.

Molecular crowding and confinement are complicated phenomena involving the interplay of entropic and enthalpic effects.^8−11^ The most common entropic effect is referred to as the excluded volume effect (“hard” interactions), which essentially states that two objects cannot be in the same place at once. The introduction of crowders in a system creates more excluded volume and is expected to increase the stability of the protein and the viscosity of the medium. The enthalpic effect includes “soft” or noncovalent interactions, which have the potential to either stabilize or destabilize the protein.^12−14^ These soft interactions can also result in the confinement of the protein. Thus, in general, crowding refers to the effects of volume exclusion due to the presence of molecular crowders, while confinement represents the restricted motions of the protein being confined within a cage-like, impenetrable boundary formed by the crowder molecules.^15,16^ Recent studies seek a more comprehensive understanding of how molecular crowding impacts protein folding, stability, aggregation, diffusion, interaction with other biomolecules, and catalysis. These studies on crowding effects vary with regard to the model system, the type of molecular crowders used, as well as their size and concentration.^17−22^

Polyethylene glycol (PEG) is one of the most commonly used synthetic crowders in crowding studies. PEG is regarded as an inert, biocompatible polymer with versatile uses throughout biological research, including drug formulation and delivery. The concept of PEGylation, a process by which PEG is attached to molecules for improved drug response, was initially described by Hoffman.^23^ The addition of PEG was found to help the molecules evade degradation by enzymes, increase solubility in water, extend the circulating half-life, and lower immunogenicity.^24^ To date, the FDA has approved over 15 PEGylated drugs.^25^ Recently, PEG molecules have also been used as a component of mRNA vaccines—both the Moderna and Pfizer-BioNTech COVID-19 vaccines use PEG 2k to help package and deliver the mRNA to cells.^26^

The physicochemical properties of PEG depend on its molecular weight (MW). For example, smaller PEG molecules are more hydrophilic, and higher MW PEG chains tend to be more amphiphilic. Changes in PEG hydrophilicity also affect how the molecule interacts with proteins.^27^ Although PEG is assumed to be an inert crowding agent, recent studies have demonstrated that PEG molecules of varying MWs impact protein structure, stability, and function differently.^28^ Different modes of interaction, such as soft noncovalent interactions versus excluded volume effect interactions between PEG and protein molecules, are observed for smaller and larger PEG molecules.^29^ As the molecular crowding effects of PEG are complex and case-dependent,^30^ exploring and understanding the effects of PEG on a wide range of proteins, including multidomain proteins, are required.

The target protein used in the present study is *Escherichia coli* (Ec) prolyl-tRNA synthetase (ProRS), a dimeric protein with identical subunits and a molecular mass of ∼127.4 kDa.^31^ Ec ProRS belongs to a diverse family of enzymes called aminoacyl-tRNA synthetases (ARSs). These enzymes catalyze the two-step reaction of attaching amino acids to their cognate tRNAs and play a vital role in protein synthesis. ProRS is a class II ARS that attaches proline to tRNA^Pro^ in a two-step reaction. The first step involves amino acid activation with ATP to form an enzyme-bound prolyl-adenylate intermediate (Pro-AMP) (eq 1). The second step involves the transfer of activated proline to the 3′-end of tRNA^Pro^, resulting in aminoacylated tRNA (Pro-tRNA^Pro^) (eq 2).
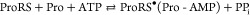
1
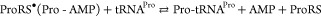
2

ProRSs are multidomain proteins, and coupled domain dynamics are crucial for maintaining catalytic efficiency.^32,33^ Earlier studies revealed that crowding agents such as PEG affect the conformation, dynamics, and catalytic function of ProRS.^34^ In particular, prolyladenylate (Pro-AMP) formation (eq 1) was affected by crowding. It was observed that the catalytic activity decreased irrespective of the size of the PEG molecules; however, the smaller MW crowding agents had a much greater impact than the larger PEG molecules.^34^ The molecular mechanism of the observed effects of PEG on Ec ProRS was unclear.

**Figure 1:**
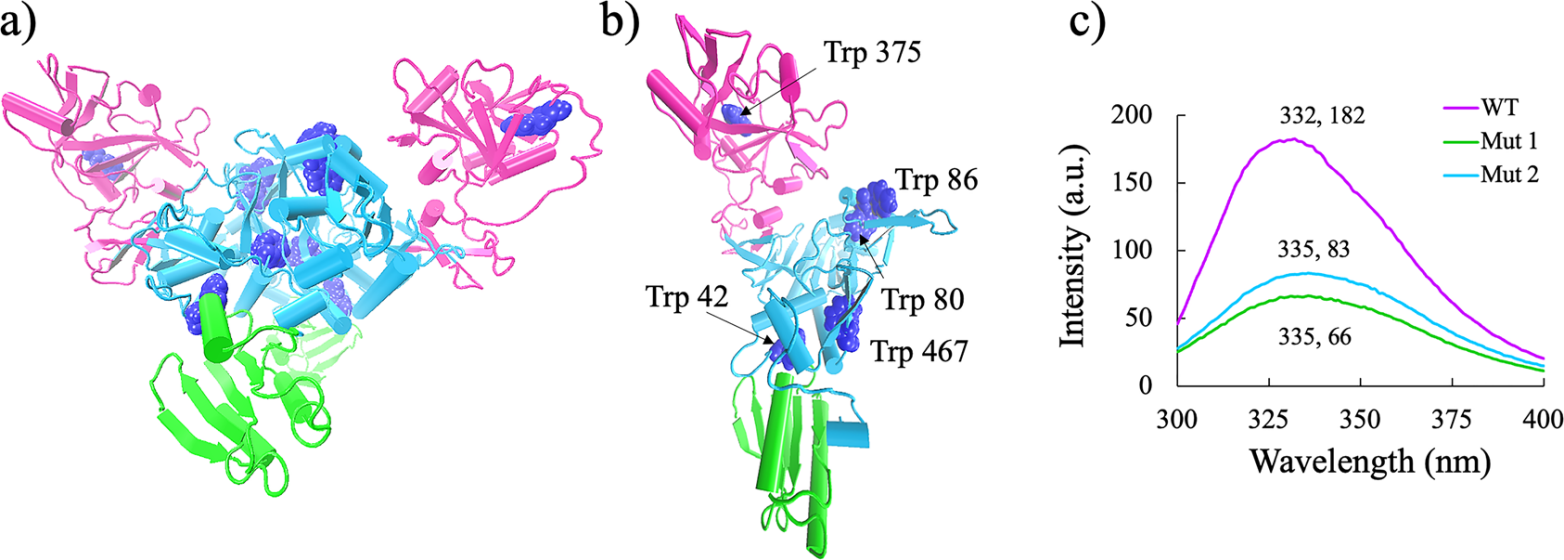
(a) Structure of dimeric Ec ProRS. The editing, catalytic, and anticodon binding domains are shown in pink, cyan, and green, respectively. The Trp residues are displayed in a blue space-filled representation. (b) Ec ProRS monomer with the same color scheme as in panel a and the five tryptophan residues labeled. (c) Fluorescence emission wavelength (nm) and intensity (a.u.) of wild-type dimeric Ec ProRS with 10 Trp residues and Mut 1 (W80FW86FW375F) and Mut 2 (W375FW467FW42F) with four Trp residues each.

In this study, we investigate whether “hard” (i.e., excluded volume), “soft” (i.e., noncovalent), or both types of interactions are at play for PEG-induced crowding/confinement of the multidomain Ec ProRS system. The present study combines experimental and computational approaches to investigate this question in more detail. Conformational changes of ProRS in the presence of various MWs and concentrations of PEG and ethylene glycol (EG) crowders were probed using spectroscopic and computational methods. In particular, intrinsic fluorescence experiments with wild-type (WT) dimeric Ec ProRS, which contains five tryptophans in each polypeptide chain (Figure 1), were conducted to explore the conformational changes in the presence of crowder concentrations varying from dilute to semidilute regimes. Three tryptophan mutant variants were designed to facilitate identification of the site(s) of conformational changes. Atomic force microscopy (AFM) was used to visualize the effects of crowders on protein structure and aggregation. Atomistic molecular dynamics (MD) simulations were performed to explore the changes in structural properties of the target protein in the crowded environment as well as to characterize the types of interactions between crowders and the target protein.

## Materials and Methods

### Materials

Proline, ATP, metal salts, and buffers were obtained from Sigma (>99% pure). EG and PEG crowders were purchased from Thermo Fisher Scientific, and [γ-[-^32^P]-ATP and [^32^P]-PP_i_ were purchased from PerkinElmer. Primers for site-directed mutagenesis and PCR were obtained from Integrated DNA Technologies.

### Expression and Purification of WT and Mutant Ec ProRS

Overexpression and purification of histidine-tagged WT and mutant Ec ProRS were performed, as described previously.^35,36^ Plasmids encoding Trp mutant variants (Mut 1: W80FW86FW375F, Mut 2: W42FW375FW467F, and Mut 3: W42FW80FW86FW467F, Figure 1) of Ec ProRS were generated by site-directed mutagenesis of pCS-M1S.^35^ Results of mutagenesis were confirmed by DNA sequencing (University of Wisconsin, Biotechnology Center-Madison). Protein expression of WT and mutant variants of Ec ProRS was induced in Ec SG13009 (pREP4) competent cells with 0.1 mM isopropyl β-d-thiogalactoside for 4 h at 37 °C. Histidine-tagged proteins were purified using Talon cobalt affinity resins and eluted with 100 mM imidazole. The purity of the proteins was evaluated by using gel electrophoresis.

### Enzyme Assays

Enzyme concentrations for all four proteins (WT and the three mutants) were determined initially using the Bio-Rad Protein Assay Kit (Bio-Rad), followed by active site titration using the adenylate burst assay.^37^

### ATP-PP_i_ Exchange Assays for Proline Activation

To evaluate the catalytic efficiencies for proline activation by the WT and mutant proteins, the ATP-PP_i_ exchange assay was performed at 37 °C according to the published method^38,39^ The concentrations of proline ranged from 0.025 to 2 mM. The enzyme concentrations used were 0.1 μM for WT and Mut 2 and 1.0 μM for Mut 1 and Mut 3. Kinetic parameters were determined from plots of velocity versus substrate concentration and fitting the data to the Michaelis–Menten equation; values represent the average of at least three determinations.

### Fluorescence Measurements

Intrinsic fluorescence spectroscopy was used to gauge the macromolecular crowding effect on ProRS. Each ProRS monomer contains five tryptophan residues, for a total of 10 residues per dimer. Since tryptophan has an excitation wavelength in the range of 280–295 nm, the enzyme was excited at 295 nm to exclude any interference from phenylalanine or tyrosine, which have excitation wavelengths close to 280 nm. The emission spectra were recorded from 300 to 400 nm. The samples included 100 mM NaCl, 30 mM phosphate buffer (pH = 7.4), 1 μM WT or mutant ProRS, and varying concentrations of the crowders, as indicated in the Figures. The final reported values were background subtracted using a no-protein sample. Fluorescence measurements were performed in a quartz cuvette with a 1 cm optical path length using an Agilent Cary Eclipse spectrophotometer. Both the fluorescence intensity and the barycentric mean wavelength (λ_bcm_), also referred to as the average emission wavelength, were determined. The latter was derived using the following method (eq 3)
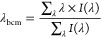
3

In the above equation, λ is the wavelength, and *I*(λ) is the emission intensity at a given wavelength. The change in λ_bcm_ [Δλ_bcm_ = λ_bcm_ (with crowders) – λ_bcm_ (without crowders)] due to the presence of crowders was examined to monitor changes in the local environment of the Trp residues.

### Melting Experiments

To probe the effect of crowding agents on the thermal stability of ProRS, melting experiments were performed with the WT enzyme in the absence and presence of crowders. Fluorescence emission spectra were obtained at 3 °C intervals from 25 to 76 °C. The melting temperature of the sample was determined by plotting the barycentric mean wavelength against the temperature. The data points were fitted to an S-curve using the Boltzmann equation on the program Origin [Origin (Pro), Version 2021, Origin Lab Corporation, Northampton, MA, USA]. Melting experiments were performed in duplicate.

### AFM Measurements

To investigate the ProRS-PEG interactions in solution, AFM measurements were performed using the Asylum Research MFP-3D AFM instrument following published protocols.^27^ The topography of ProRS-PEG complexes was probed using the dynamic tapping mode with a cantilever force constant of 0.15–0.55 N/m to avoid denaturation of proteins while allowing the analysis of the height of the samples under dilute and crowded conditions. Briefly, protein samples were prepared using 1 μg/mL ProRS and 100 μg/mL PEG 8k or 20k in 30 mM phosphate buffer (pH = 7.4) and 100 mM NaCl. Samples were first incubated for 30 min to allow for any chemical interactions to occur between PEG and ProRS. Samples were then applied to freshly cleaved highly oriented pyrolytic graphite (HOPG), a hydrophobic and atomically flat surface, and incubated for an additional 30 min. The samples were then rinsed to remove salts using 20 aliquots of 100 μL of ddH_2_O applied via micropipette, followed by drying in vacuo for 30 min to ensure the removal of all water on the HOPG surface. Samples deposited on HOPG were scanned at a rate of 0.4 Hz with parameters of 512 scan lines and 512 scan points. The lower concentration of PEG crowders was chosen for AFM experiments because PEG self-aggregates into sheets on HOPG at higher concentrations,^40^ which interferes with the detection of ProRS-PEG complexes.

### MD Simulations

All atomistic simulations were carried out using the NAMD program^41,42^ (version 2.13) and the CHARMM program suite^43^ on a 61-node (3904 cores) BOSE cluster at the Blugold Center for High-Performance Computing, UW-Eau Claire. The CHARMM36 all-atom force field^43−45^ and CHARMM36 parameters were used for all molecular mechanical calculations and MD simulations. Electrostatic interactions were modeled using the particle mesh Ewald method.^46,47^ Nonbonding interactions were modeled using a switching function with a “switchdist” of 9 Å, a cutoff of 10 Å, and a “pairlistdist” of 16 Å. The leapfrog Verlet algorithm^48^ was employed for integration, and a time step of 2 fs was used to compute atomic velocities and displacements. A modified Nosé–Hoover method^49,50^ was employed during constant-pressure MD simulations, where pressure fluctuations in the barostat were controlled using Langevin dynamics.^51,52^ A periodic boundary condition was used, which controls the pressure by dynamically adjusting the unit cell volume and rescaling the atomic coordinates. The sampled conformations constitute an isothermal–isobaric (*NPT*) ensemble, which yields enthalpic changes.

The three-dimensional structure of Ec ProRS was generated by homology modeling, with the crystal structure of *Enterococcus faecalis* ProRS (PDB code: 2J3L) used as a template.^34^ Visualization of all molecular structures, measurement of distances, and calculation of radii of gyration were carried out using the Visual Molecular Dynamics (VMD)^53^ program. A homemade script was used for adding hydrogen to the protein by maintaining the charges of acidic and basic amino acid residues in their protonated states at pH 7.0. The protonation state and the location of protons of the histidine residues were determined through the computation of the p*K*_a_ using the Propka application of PDB2PQR.^54^ Additionally, each protein subunit was labeled with a specific segment identifier.

All simulations involved the dimeric Ec ProRS. They were carried out in water (i.e., in dilute conditions), EG, and PEGs (MW of 600, 8k, and 20kDa). Each simulation system consisted of an assembly of dimeric proteins, requiring numbers of crowders, water molecules, and ions. Before the addition to the assembly, the Ec ProRS dimer was equilibrated using a 2 ns MD simulation. Similarly, individual EG and PEG 600 crowder molecules were geometrically optimized in the gas phase using 200 and 1000 steps of the Newton–Raphson optimizer method available in the CHARMM program suite, respectively. The PEG 8k and 20k crowding agents were allowed to partially fold by running 500 ps of equilibration dynamics in water, as previously described.^40^

For EG and PEG 600 crowders, Packmol^55^ was used to create the solvated protein-crowder assembly by randomly distributing crowders as well as solvent molecules around the dimeric Ec ProRS. For PEG 8k and PEG 20k systems, the assembly was generated by placing the crowders in close proximity to the target protein, which could maximize the possibility of protein-crowder interactions. All structures were explicitly solvated (with the TIP3P model)^56^ and ionized (with sodium atoms) with VMD plugins. Once generated, the neutralized solvated protein-crowder assembly was minimized using 50,000 steps of the conjugate gradient method.

Two different types of computational experiments were carried out to examine the effects of crowding and confinement. The first set of experiments was performed with EG, PEG 600, and PEG 8k systems, where the same mass ratio of the protein to the total number of EG units was maintained. The second set of experiments was conducted with PEG 20k, in which the mole ratio of PEG to Ec ProRS dimers was 1:1 or 1:5.

#### Root Mean Square Deviation

The extent of change in protein conformation during the 100 ns MD simulation was assessed by monitoring the deviation of each frame from the starting structure obtained after optimizing the solvated protein system. The per-frame RMSD was calculated from the square root of the mean square of the deviations averaged over all C_α_ atoms for a specific frame using eq 4
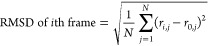
4where *N* is the number of C_α_ atoms, *r*_*i*,*j*_ is the position vector for the *j*th C_α_ atom observed in the *i*th frame, and *r*_0,*j*_ represents the position vector of the *j*th C_α_ atom for the ProRS structure at the beginning of the simulation (i.e., the zeroth frame). The RMSD is plotted with respect to the length of the simulation.

#### Root Mean Square Fluctuation

The flexibility of the backbone of the Ec ProRS was examined by computing the per residue RMSF using all conformations generated during the 100 ns MD simulation for a specific C_α_ atom of the residue (eq 5)
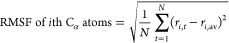
5where *N* is the number of conformations, and *r*_*i*,*t*_ and *r*_*i*,av_ represent the position vector for the *i*th C_α_ atom at time *t* and averaged over all conformations, respectively.

The MWs and concentrations of PEG molecules used in various experiments and MD simulations are listed in Table 1. The physical properties of synthetic crowder solutions, including PEG solutions, change with concentration.^57^ At low concentrations, polymers act like individual molecules; however, they interact with each other at concentrations above a so-called overlap concentration.^58^ The overlap concentration of an aqueous solution of a polymer is the critical concentration at which the polymer chains start to overlap and form meshlike networks and, as a consequence, interfere with any measurements involving concentration or size variations. To mimic the intracellular concentration of crowders, 100 mg/mL PEG was used for most experiments in the present study, which is above the overlap concentration for PEG 8k and 20k.

**Table 1:** List of Sizes and Concentrations of Polyethylene Glycol Used in Experiments and MD Simulations in the Present Study

study	PEG size	concentration (mg/mL)	dilute/semidilute regime
MW variation	EG, 200, 400, 600, 1k, 2k, 4k, 8k, 20k	50	dilute
concentration variation	600, 2k	25–400	dilute
	8k, 20k	25–100	dilute to semidilute
Trp mutants	8k	100	semidilute
melting experiments	EG, 600	200	dilute
	8k	50	dilute
	8k	200	semidilute
AFM	20k	0.100	dilute
MD simulations	EG	83	dilute
	600	83	dilute
	8k	35	dilute
	20k	10	dilute

## Results and Discussion

### Impact of Mutations on Proline Activation Efficiency

The catalytic efficiency of WT Ec ProRS and three Trp mutant variants was examined using the ATP–PP_i_ exchange reaction to monitor amino acid activation (eq 1). These mutations were designed to eliminate Trp residues in different combinations to probe the sites of conformational change. Simultaneous Trp to Phe substitution at positions 80, 86, and 375 (Mut 1) resulted in an ∼7-fold decrease in *k*_cat_/*K*_M_, while a ∼5-fold decrease was observed in the case of Mut 2 containing Trp to Phe substitutions at positions 42, 375, and 467 (Table 2). Mut 3, which contained four Phe to Trp substitutions, was inactive and was not investigated further. Mut 1 and Mut 2 were used as probes to identify the sites of conformational changes in the presence of crowding agents, as discussed in the next section.

**Table 2:** Catalytic Efficiency of Proline Activation and Fluorescence Properties of WT Ec ProRS and Trp Mutant Variants^a^

ProRS	*k*_cat_/*K*_M_ [1/(min × μM)]	fold decrease in *k*_cat_/*K*_M_	% decrease in fluorescence intensity
WT	2.9 ± 0.26	1	11.8
Mut 1	0.44 ± 0.27	6.6	9.7
Mut 2	0.59 ± 0.12	4.9	11.4
Mut 3	N/A	not active	N/A

a Kinetic experiments were performed in triplicate, with the standard deviation indicated. The last column indicates the percent decrease in Trp fluorescence intensity in the presence of 100 mg/mL PEG 8k relative to protein alone.

### Conformational Changes Observed through Intrinsic Fluorescence Measurements

The intrinsic fluorescence intensity of a protein reflects its interactions with the surrounding environment. The fluorescence emission is reduced when quenching occurs either due to collisions with molecules in the excited state (hard interactions and dynamic quenching) or due to complex formation with molecules in the ground state (soft interactions and static quenching). The change in the barycentric wavelength reflects how exposed the Trp residues are to the surrounding polar water molecules. A decrease in wavelength (blue shift) indicates the Trp is less exposed to water, suggesting a transition toward a more compact conformation. An increase in wavelength (red shift) indicates an increase in exposure of Trp to the solvent, suggesting a less compact conformation. Intrinsic fluorescence measurements were performed to evaluate the effects of crowding agents on protein conformation by monitoring the change in tryptophan fluorescence intensity and emission wavelength.

#### Effects of PEG Size on Protein Conformation

The effect of variable-sized PEG molecules on the conformation of WT Ec ProRS was examined using nine crowders of different MWs ranging from the EG monomer to PEG 20,000 (Figure S1). Similar amounts of each crowding agent were used. In the dilute regime, i.e., below the overlap concentration of PEGs (Table S1),^58^ the smaller PEG molecules had little to no effect on the fluorescence intensity, but the larger sizes (8k and 20k) induced statistically significant quenching (Figure S1a); the 90% confidence intervals computed for the relative fluorescence intensity of Ec ProRS in the presence of PEG 8k and PEG 20k were 0.84 ± 0.03 and 0.74 ± 0.07, respectively. PEG molecules up to 8k had no effect on λ_bcm_ (eq 3); however, both PEG 20k and a PEG cocktail (*C*) indicated a slight increase in emission wavelength (Figure S1b), suggesting that the largest PEG crowder may have induced a conformational change that caused Trp residues to be more solvent exposed. A similar observation was made when the effects of PEG molecules of variable MW were investigated on bovine serum albumin (BSA) by Lai et al.^28^ It was reported that PEG molecules with MWs larger than BSA have a greater impact on protein conformation than smaller PEGs; low-MW PEGs do not exhibit any effects.

#### Effects of the PEG-to-Protein Ratio on Protein Conformation

**Figure 2:**
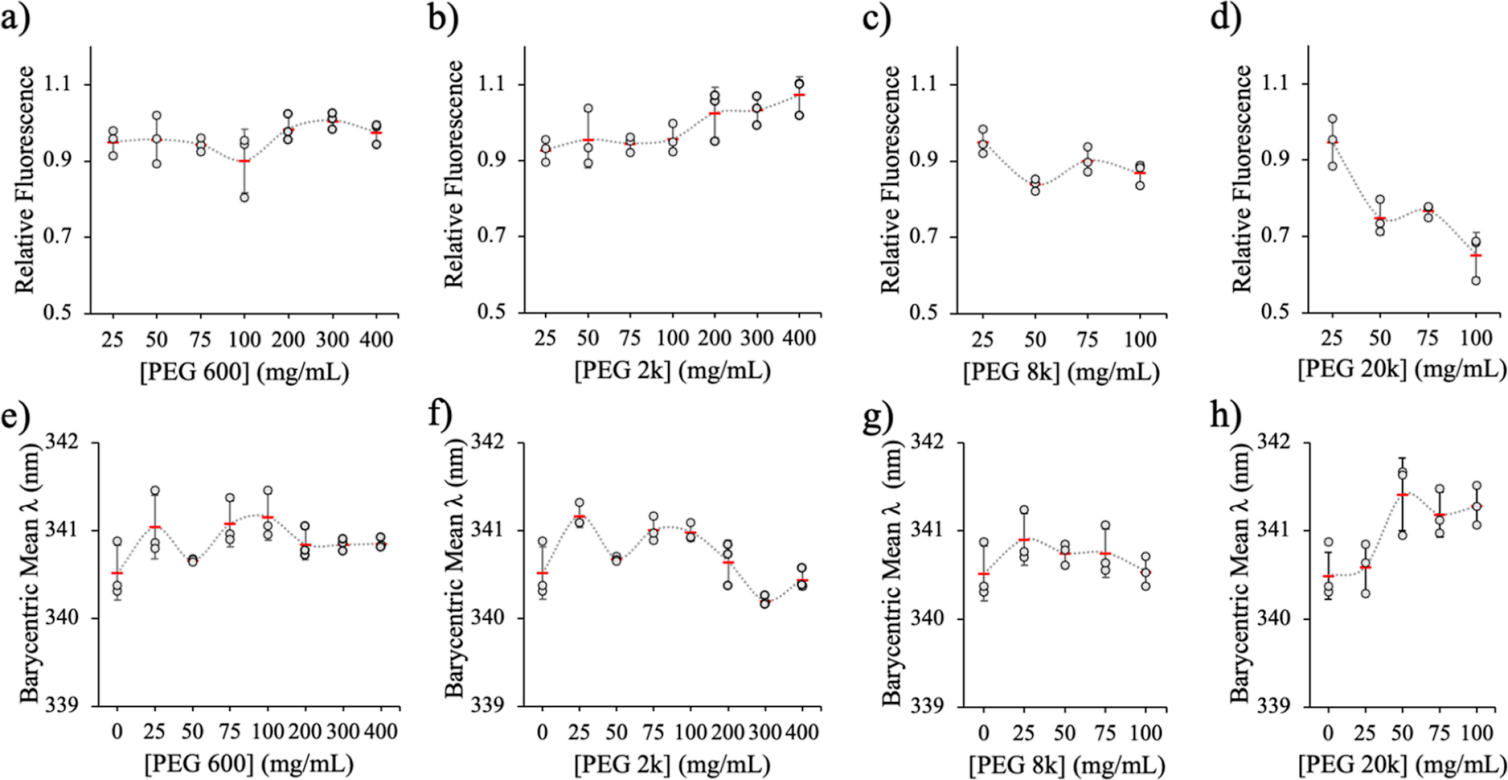
Impact of PEG concentration on fluorescence of WT Ec ProRS. PEG sizes vary from a MW of 600 to 20k. (a–d) Relative fluorescence of samples containing various concentrations of PEGs as compared to protein alone, which was set to 1.0. (e–h) The barycentric mean wavelength in the presence of various concentrations of PEG600, 2k, 8k, and 20k. The results presented here are an average of three trials, with the mean (horizontal red lines) and standard deviation (vertical black lines) indicated. The smooth lines join the mean values in each case.

We next measured the concentration-dependence of PEG crowders on the conformation of Ec ProRS using PEG 600, 2k, 8k, and 20k (Figure 2). No significant impact on either the relative fluorescence intensity or λ_bcm_ in the presence of PEG 600 and 2k was observed at concentrations up to 100 mg/mL (Figure 2a,b,e,f). Even at very high concentrations (200–400 mg/mL), no changes in the fluorescence properties of ProRS were observed in the presence of PEG 600 (Figure 2a,e). However, under these conditions, PEG 2k resulted in a modest enhancement in fluorescence intensity but no significant change in λ_bcm_ (Figure 2b,f). PEG 8k resulted in a significant decrease (∼12%) in fluorescence at the lower concentrations, but no impact on the emission wavelength was detected (Figure 2c,g). PEG 20k had by far the largest impact on the ProRS fluorescence. Increasing concentrations of PEG 20,000 from dilute to semidilute conditions (Table S1)^57,58^ caused a significant decrease (∼50% at 100 mg/mL relative to protein alone) in the Trp intrinsic fluorescence (Figure 2d), indicating conformational changes in the presence of this crowder. The change in λ_bcm_ in the presence of PEG 20k was modest (Figure 2h). Based on these data, we infer that the protein conformation was affected in the presence of large MW PEG crowders, i.e., PEG 8k and 20k.

#### Mutational Studies to Identify the Sites of Conformational Changes

The effects of PEG 8k on the fluorescence properties of two Ec ProRS mutant proteins were studied. Mut 1 and Mut 2 contain only four Trp residues compared to ten in the WT enzyme (Figure 1a–c). The ∼2:5 Mut 1/2 to WT ratio of fluorescence intensity measured was consistent with the elimination of 6 Trp residues in the mutants (Figure 1c). In the presence of 100 mg/mL PEG 8k, similar decreases in Trp fluorescence intensity were observed for WT and mutant proteins (Table 2). PEG 8k was chosen here instead of PEG 20k because the commercially available PEG 20k contains fluorescent stabilizers such as 3-*tert*-butyl-4-hydroxyanisole and thus fluoresces.^40^ PEG 20,000 interfered with the fluorescence measurements with mutant variants due to their weaker fluorescence properties relative to the WT enzyme. Mut 1 has two remaining Trp residues (W42 and W467) in the catalytic domain (CD), proximal to the anticodon-binding domain (ACB), whereas Mut2 maintains CD domain residues W80 and W86 located on the opposite end of the CD, proximal to the editing domain (ED). The comparable changes in fluorescence intensity in the presence of PEG 8k for Mut 1 and Mut 2 suggest that both regions of CD, one proximal to ED and the other at the interface of CD and ABD, were equally affected by the crowder molecules. The effect of PEG 8k crowders on the highly flexible ED was not studied experimentally as Mut 3 was catalytically inactive (Table 2). The SASA analysis, however, suggested that W375 in ED was affected by the crowder molecules, which, in turn, indicates that ED was also impacted by PEG molecules (vide infra).

### Evidence of Alteration in Protein Stability Due to PEG Crowders from Melting Experiments

Adams et al.^34^ have shown that the catalytic efficiency of Pro-AMP synthesis by Ec ProRS (eq 1) decreased in the presence of PEG molecules, which could be due to conformational changes. The hard (volume exclusion, entropic) and soft (noncovalent, enthalpic) interactions by polymeric crowders can stabilize or destabilize protein structures, making them either compact or elongated.^59^ The thermal stability of WT Ec ProRS was tested by performing melting experiments in the absence and presence of crowders. The melting temperature (*T*_M_) of Ec ProRS (1 μM) was determined in the presence of EG, PEG 600, and PEG 8k. EG (200 mg/mL) and PEG 8k (50 mg/mL) have no impact on the *T*_M_ of Ec ProRS, whereas PEG 600 (200 mg/mL) and PEG 8k (200 mg/mL) have a stabilizing effect on the protein (Table 3, Figure S2). *T*_M_ was significantly higher (53.6 ± 0.4 °C) in the presence of PEG 8k crowders compared to that in the absence of crowders (48.9 ± 0.5 °C), suggesting a crowder-induced impact on ProRS structure and stability. This observation has been corroborated with MD simulation data, where PEG polymers induced a compact “closed” conformation of ProRS dimer (vide infra).

**Table 3:** Melting Temperature of Ec ProRS in the Presence of PEG Crowders^a^

no crowders	ethylene glycol (EG) (200 mg/mL)	PEG 600 (200 mg/mL)	PEG 8k (50 mg/mL)	PEG 8k (200 mg/mL)
48.9 ± 0.5 °C	48.8 ± 0.2 °C	50.6 ± 0.4 °C	48.4 ± 0.2 °C	53.6 ± 0.4 °C

a The concentration of Ec ProRS was 1 μM. The fluorescence emissions were collected at 3 °C intervals from 25 to 76 °C with an excitation wavelength of 295 nm. The melting temperatures were determined by plotting the barycentric mean wavelength against the temperature (Figure S2). Experiments were performed in triplicate, with the standard deviation indicated.

Midsize PEG molecules are capable of soft interactions but have a variety of effects, including stabilization or destabilization and favoring or disfavoring aggregation.^60−64^ In an earlier study, PEG 8k was found to have a destabilization effect on chemotaxis protein Y (CheY)^65^ and thus resulted in a decrease in melting temperature. It was reported that the noncovalent interactions with protein side chains induce conformational change and destabilization in CheY. Similarly, PEG 35k was found to have a destabilizing effect on human serum albumin and assist in the denaturation process.^66^ Other recent findings suggest that larger PEG molecules stabilize proteins due to the excluded volume effect.^58,59,61,64^ The melting experiment in the presence of 200 mg/mL PEG 8k was carried out above its overlap concentration (Table S1)^58^ where synthetic polymer crowding agents can form mesh-like networks.^67,68^ The trapping of protein molecules in a mesh-like network of polymers likely induces structural stabilization. Hard interactions can also drive proteins toward compact structures, resulting in an increase in the melting temperature. The increase in *T*_M_ of Ec ProRS indicates that PEG has a distinct effect on the each protein system.

### Evidence of Protein Aggregation in Crowded Environments from Atomic Force Microscopy

**Figure 3:**
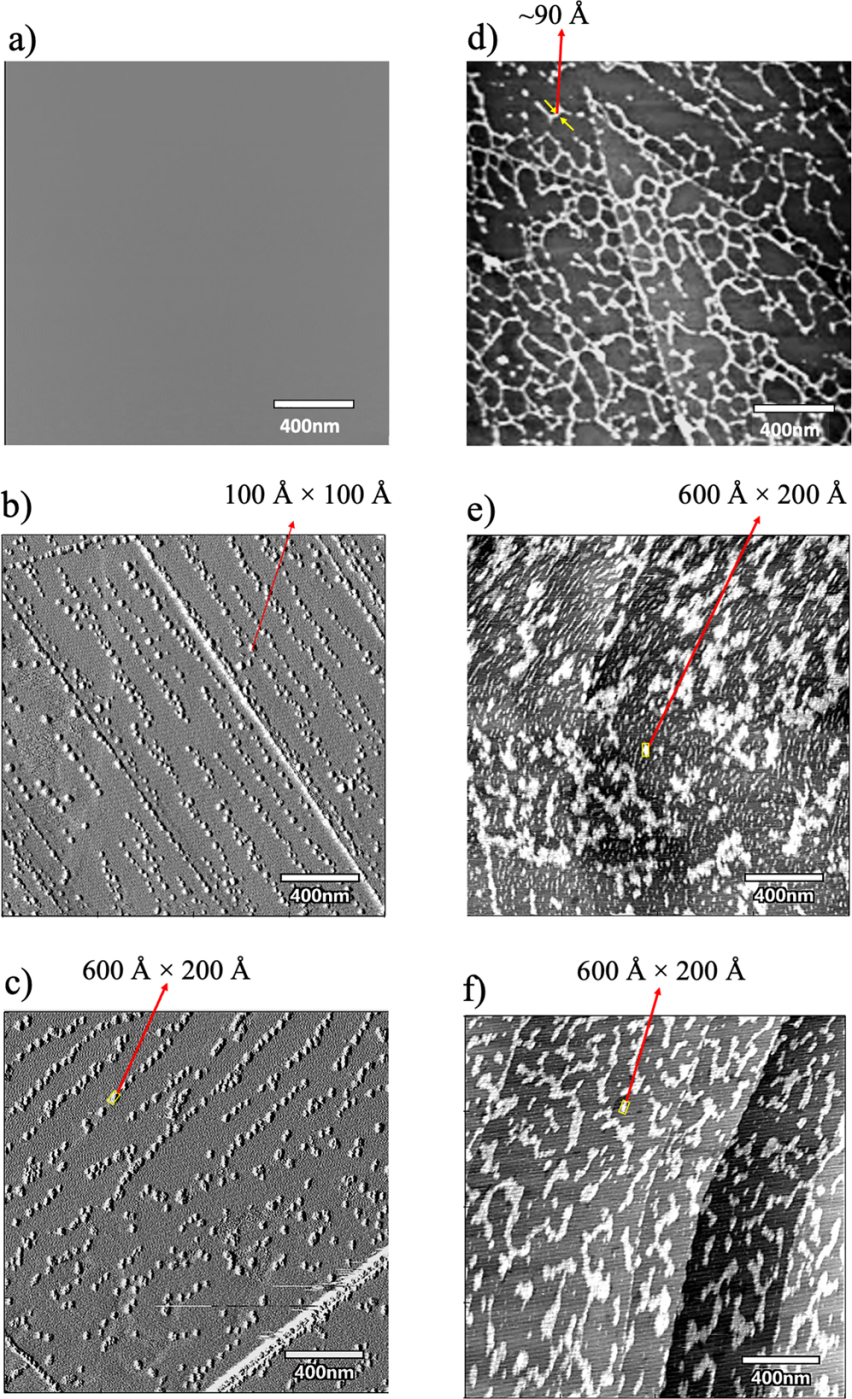
AFM images of WT Ec ProRS in different conditions: (a) HOPG substrate; (b) 100 μg/mL PEG 8k; (c) 100 μg/mL PEG 20k; (d) 1 μg/mL WT Ec ProRS; (e) 1 μg/mL WT Ec ProRS in 100 μg/mL PEG 8k; and (f) 1 μg/mL WT Ec ProRS in 100 μg/mL PEG 20k.

AFM experiments were carried out to explore the topographic change of WT ProRS in the presence of PEG 8k and PEG 20k (1:100 protein/PEG ratio). These larger MW PEGs are known to be amphiphilic, resulting in the confinement of proteins.^27^ The AFM images revealed a uniform surface for the HOPG substrate (Figure 3a). On the other hand, both PEG 8k and PEG 20k crowders formed self-aggregates on the HOPG surface (Figure 3b,c). The PEG 8k clusters were smaller in size with a surface area of ∼100 × 100 Å^2^ (Figure 3b), while the PEG 20k formed larger clusters with a surface area >600 × 200 Å^2^ (Figure 3c). The formation of PEG clusters in aqueous solution is consistent with the earlier-reported MD simulation results for PEG 20k^40^ as well as in this study (vide infra). In contrast, the Ec ProRS molecules were found to be deposited uniformly on the HOPG surface as worm-like expanded structures in the absence of crowders (Figure 3d). The thickness of these worm-like tubes ranged between 80 and 100 Å, like the width of the Ec ProRS dimer. In the presence of PEGs, larger clusters were observed (Figure 3e,f), indicating PEG-protein interactions. These ProRS-PEG aggregates are formed either by multiple PEG molecules encapsulating one or more ProRS dimers^27^ or by several ProRS dimers discretely interacting with chains of PEG 8k or PEG 20k.^28^ A thorough molecular dynamics simulation study was conducted to gain a molecular-level understanding of these ProRS-PEG interactions.

### Impact of Crowding on Protein Dynamics as Observed through Molecular Dynamic Simulations

The effects of crowding on the structure and conformational dynamics of bacterial ProRSs^34^ and other enzyme systems were noticed earlier.^66,69−71^ For bacterial ProRS, coupled-domain dynamics are critical for maintaining catalytic efficiency;^32^ anticorrelated motion of the proline-binding loop (PBL) with respect to ED is crucial for substrate binding. ED dynamics leads to conformational preorganization, which was previously shown to contribute to about half of the catalytic power of the Ec ProRS synthetic active site.^33^ The global dynamics of the ED modulate the fluctuations of active site residues; the local fluctuations of active site residues impact the height and width of the Gibbs activation energy profile by fine-tuning the substrate orientation to facilitate reactive collisions. PEG crowders can impact the intrinsic dynamics as well as the “open” to “closed” conformational transition in Ec ProRS. To gain insight into the effects of PEG crowding on Ec ProRS dynamics, the overall conformational change (global), residuewise fluctuations (local), and shifts in the conformational ensemble were examined in the presence of crowders. The 100 ns MD simulation data were used to calculate the RMSD (eq 4) of conformational evolution, the RMSF (eq 5) of the backbone, and the “open” to “closed” conformational transition. The soft interactions between PEG and protein side chains were assessed through the solvent-accessible surface area (SASA) of the 10 Trp residues.

#### Clustering of PEGs and PEG-Protein Interactions as Revealed in the Simulated Models

MD simulations of Ec ProRS in the presence of EG, PEG 600, PEG 8k, and PEG 20k were performed. These polymeric crowders were chosen because PEG 600 had the greatest impact on enzyme kinetics,^34^ and PEG 8k and 20k had a significant impact on protein conformational change (Figures S1 and S2). The details of the ternary systems containing Ec ProRS dimer, water, and cosolutes developed for 100 ns MD simulations are provided in Table 4. The protein atom/crowder atom ratio was ∼1:1.3 for EG, PEG 600, and PEG 8k; the simulation box was slightly larger for the PEG 8k system to accommodate 18 of the crowder molecules. For simulation with five Ec ProRS dimers in the presence of a PEG 20k molecule, the simulation box was required to be much larger to accommodate all atoms (Table 4).

**Table 4:** System Parameters Used for 100 ns MD Simulations of Dimeric Ec ProRS in Dilute and Crowded Environments

system (crowders + ProRS dimers)	protein atoms	water atoms	sodium ions	crowder atoms	dimension of the orthorhombic box (Å^3^)
dilute^a^	17,693	263,907	42	0	156 × 152 × 125
EG	17,693	216,654	42	23,269	160 × 150 × 120
PEG 600	17,693	216,654	42	23,030	160 × 150 × 120
PEG 8k	17,693	649,578	42	23,112	198 × 178 × 196
PEG 20k	17,693	300,327	42	3174	160 × 158 × 133
PEG 20k + five ProRS dimers	88,465	941,055	210	3174	340 × 151 × 210

a ProRS system in the absence of crowder molecules.

**Figure 4:**
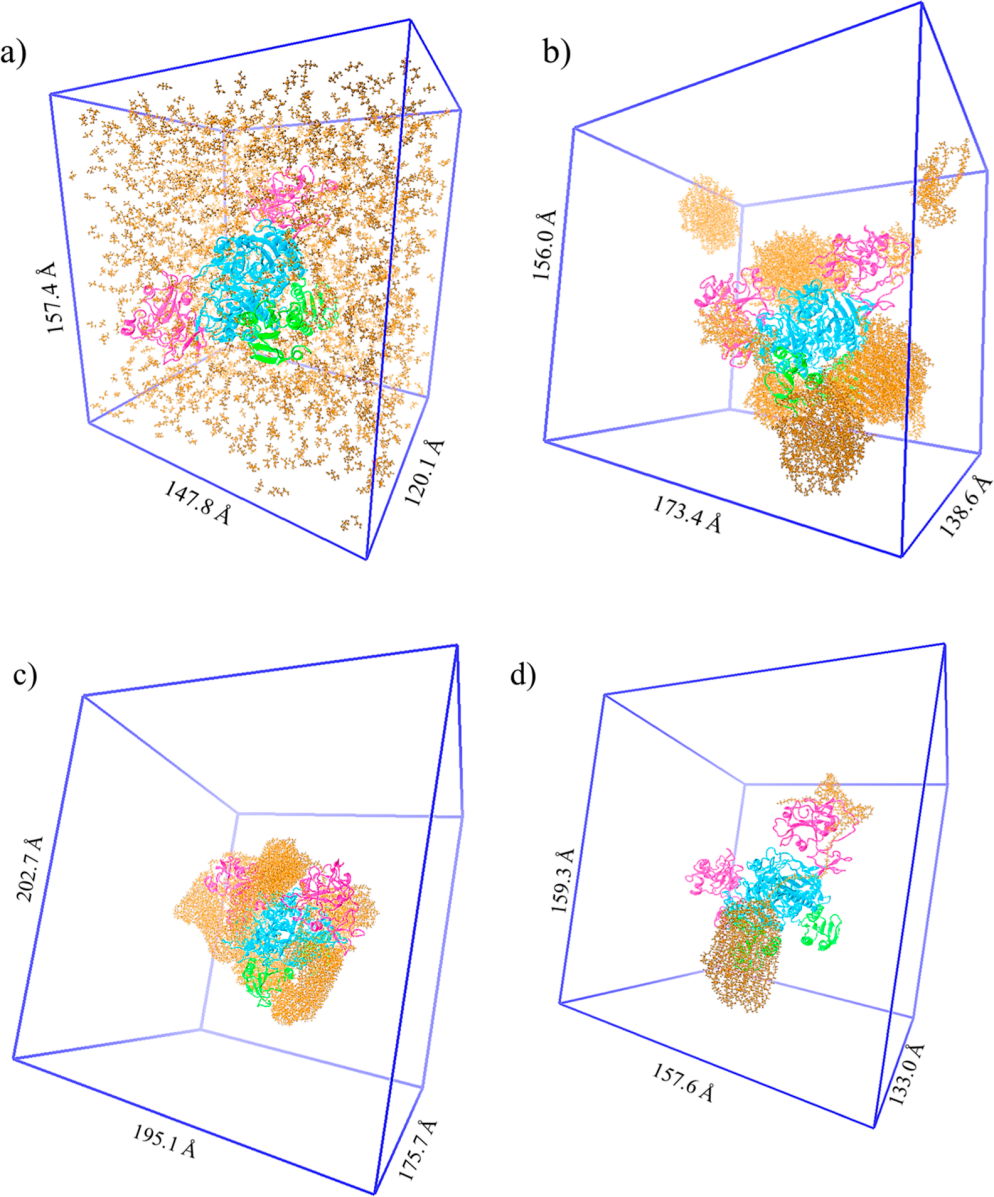
Dimeric Ec ProRS in a simulation box containing (a) EG, (b) PEG 600, (c) PEG 8k, and (d) PEG 20k. These images were obtained after 100 ns of MD simulations with explicit solvation; water molecules were omitted for clarity.

**Figure 5:**
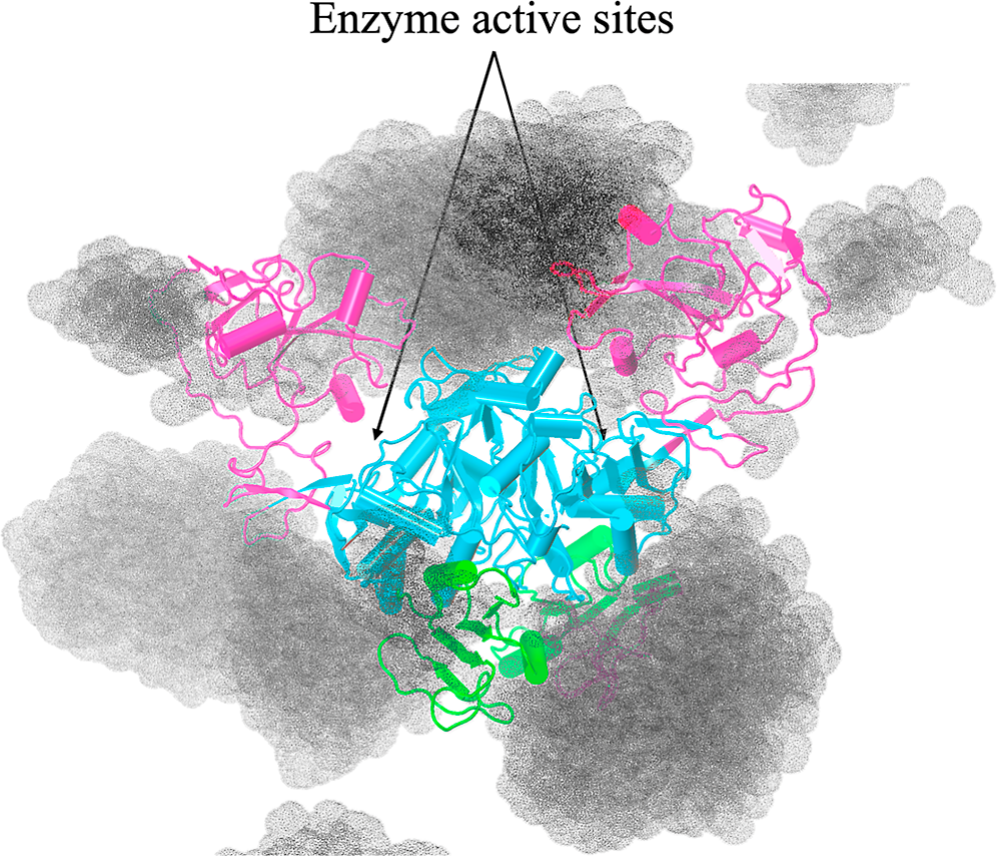
Blocking of the active sites of Ec ProRS by PEG 600 clusters. The dotted spheres represent the solvent-accessible surface of the PEG 600 molecules, calculated using a probe radius of 1.4 Å.

The arrangement and interactions of crowder molecules among themselves and with the protein side chains were investigated after 100 ns simulations (Figure 4). The evolution of these protein systems during the simulation revealed that the monomer EG molecules were uniformly distributed all over the protein and appeared to have less impact on the Ec ProRS conformational dynamics (Figure 4a). PEG 600 molecules formed clusters through self-aggregation and were observed to alter their sizes during the simulated dynamics. This is consistent with the behavior of PEG molecules in an aqueous solution, as reported earlier.^40^ The fluidity of the aggregates was apparent as splinters of isolated PEG 600 molecules were found to leave one cluster to join the other, as revealed from the variable sizes of PEG 600 clusters in Figure 4b. One large cluster consistently occupied the interspatial region of the two EDs, which effectively blocked the active sites of the Ec ProRS dimer (Figure 5).

The larger PEG polymers (PEG 8k and 20k) formed a cage-like assembly, encapsulating the dimeric Ec ProRS (Figure 4c,d). As reported earlier, similar aggregates were observed in the aqueous simulation of PEG 20k.^40^ The 18 PEG 8k molecules self-assembled, forming compact clusters that wrapped around all three domains of the dimeric Ec ProRS (Figure 4c). These clusters also cover the substrate-binding pockets of both subunits. However, being larger in size, the blockade is less effective compared to what was observed for PEG 600 clusters. PEG 20,000 showed a similar tendency, where a large portion of the polymer formed a cluster through self-interactions, while a smaller segment of it wrapped around the Ec ProRS, especially one of its EDs (Figure 4d). This resulted in a difference in the motion of the two EDs in the presence of PEG 20k, which was observed in RMSD and RMSF studies (vide infra).

#### Evidence of Conformational Rigidity Due to Crowding by PEG Molecules

**Figure 6:**
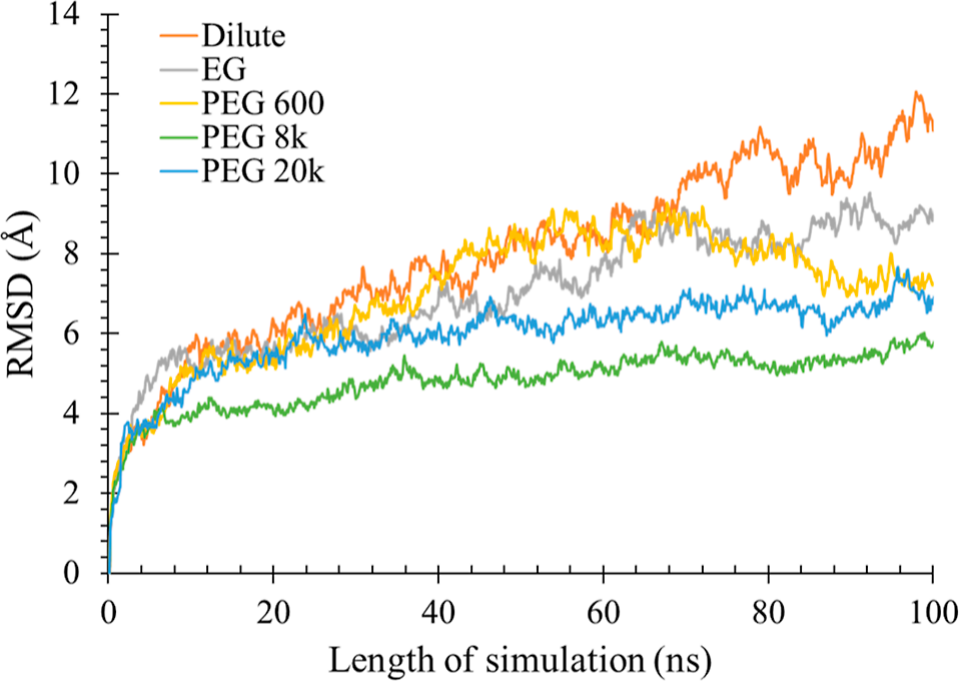
RMSD plots for the backbone atoms of Ec ProRS in the absence and presence of PEG crowders.

The overall conformational change of the dimeric Ec ProRS under dilute and crowded conditions was assessed by computing the RMSD for the protein along the path of simulation. The DCD trajectory files obtained from the 100 ns simulations were analyzed, and RMSD values from the starting equilibrated conformation were calculated (eq 4). As shown in Figure 6, a rapid change in RMSD from the starting conformation was noted between 0 and 10 ns, after which the change in RMSD was reduced. The dimer underwent a significant conformational change; the RMSD varied up to ∼12 Å with respect to the starting conformation in the absence of crowders. The RMSD was slightly less (∼9 Å) in the presence of EG, while the PEG 600, PEG 8k, and PEG 20k systems showed averaged RMSD values ranging from 6 to 7 Å. These simulation data suggested that the crowder molecules impacted the overall conformational flexibility of the dimeric Ec ProRS.

#### Radius of Gyration and Interediting Domain Separation

To further investigate if PEG crowders impact the compactness of the dimeric Ec ProRS, the radius of gyration of the dimeric protein was computed. The PEG crowders indeed had an impact on the protein rigidity; the radius of gyration decreased by 4–5 Å in the presence of PEG crowders; EG had less effect in altering the compactness of Ec ProRS (Table 5).

**Table 5:** Conformational Ensemble Shifts Observed After 100 ns Simulations due to the Presence of Various PEG Crowders^a^

	radius of gyration (Å)	distance between the COMs of ED (Å)	open (%)		closed (%)	
protein crowder system	ProRS dimer	ProRS dimer	ProRS subunit A	ProRS subunit B	ProRS subunit A	ProRS subunit B
dilute^b^	40.3	103.6	79	73	21	27
EG	38.4	88.6	67	87	33	23
PEG600	35.5	70.2	0	0	100	100
PEG 8k	35.5	57.1	0	7	100	93
PEG 20k	35.9	70.9	0.6	92	98	8

a “Open” conformations are defined by a Q88(C_a_)–P318(C_a_) distance greater than 15 Å, while “closed” conformations have a Q88(C_a_)–P318(C_a_) distance less than or equal to 15 Å. The distance between the COMs of two EDs at the starting conformation was 80 Å.

b ProRS system in the absence of crowder molecules.

**Figure 7:**
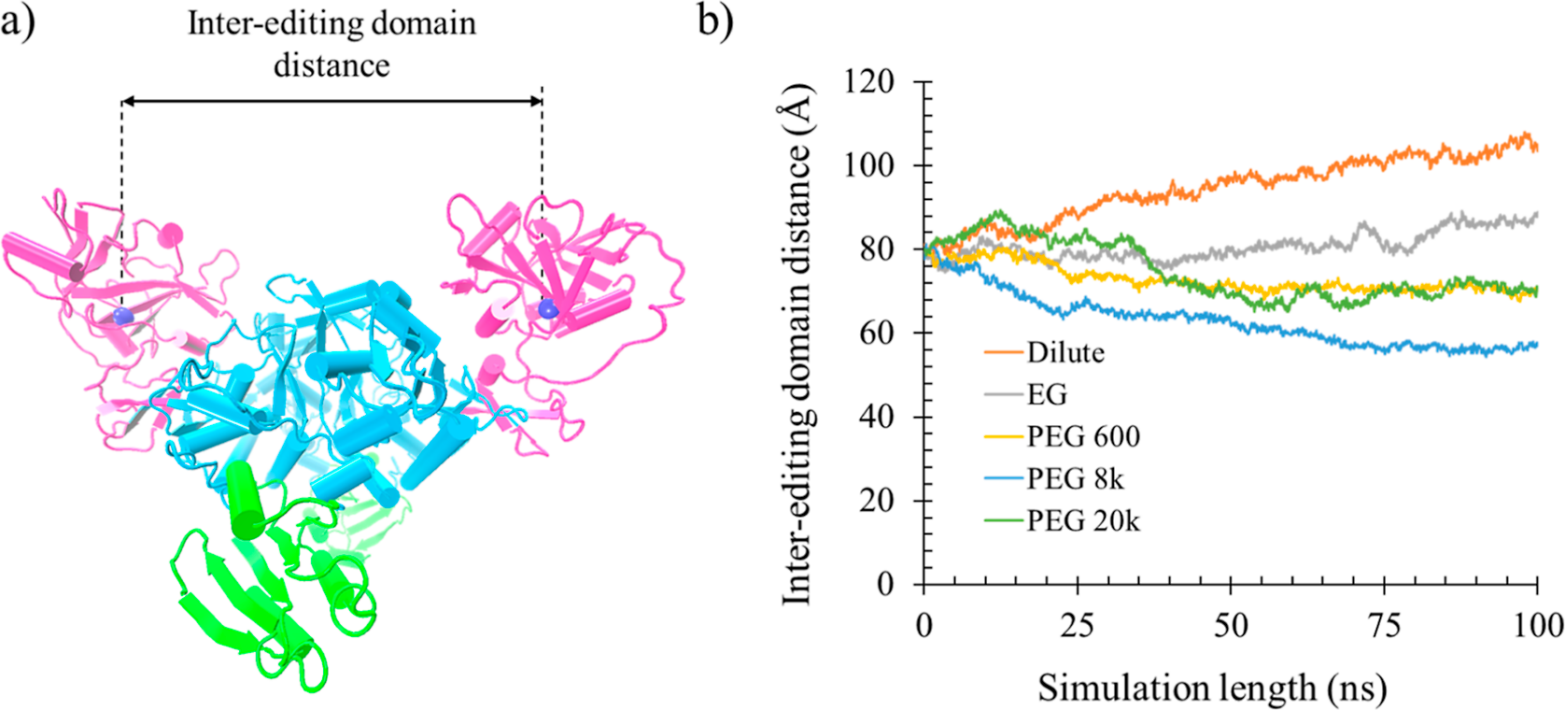
Changes in Ec ProRS domain dynamics due to various PEG crowders. (a) Dimeric Ec ProRS with the center-of-mass (COM) of each editing domain (residues 224–407, pink) shown as a blue sphere. (b) Variation of the distances between editing domain COMs plotted against the length of the simulation.

An analysis of the inter-editing domain separation was also carried out by measuring the distance between the centroids of the two EDs over a 100 ns simulation. The C_α_ of the S298 was within 2 Å of the computed center of mass (COM) of ED (residue 224–407) and was used as the centroid of ED in all calculations (Figure 7a). The distance of separation between the two COMs of the EDs was plotted as a function of the simulation time (Figure 7b). In the absence of crowders, i.e., in dilute condition, the inter-editing domain distance increased from 80 to 104 Å, i.e., 24 Å, during the 100 ns simulation (Table 5). The separation between the two COMs increased slightly (∼9 Å) in the presence of EG. Interestingly, the two EDs moved close to each other in the presence of larger PEGs; the interediting domain distance shrank by 10, 23, and 9 Å for the PEG 600, 8k, and 20k crowders, respectively (Table 5 and Figure 7). The smaller impact observed for the PEG 20,000 crowder is because only one PEG 20,000 crowder was used in the simulations. These simulation results in the presence of larger MW PEG crowders are indicative of the crowder-induced compactness of the Ec ProRS structure. A similar observation, i.e., the decrease in distance between the two EDs of Ec ProRS, was also made earlier for monomeric crowders.^34^

#### Ratio of Open-To-Closed Conformations

**Figure 8:**
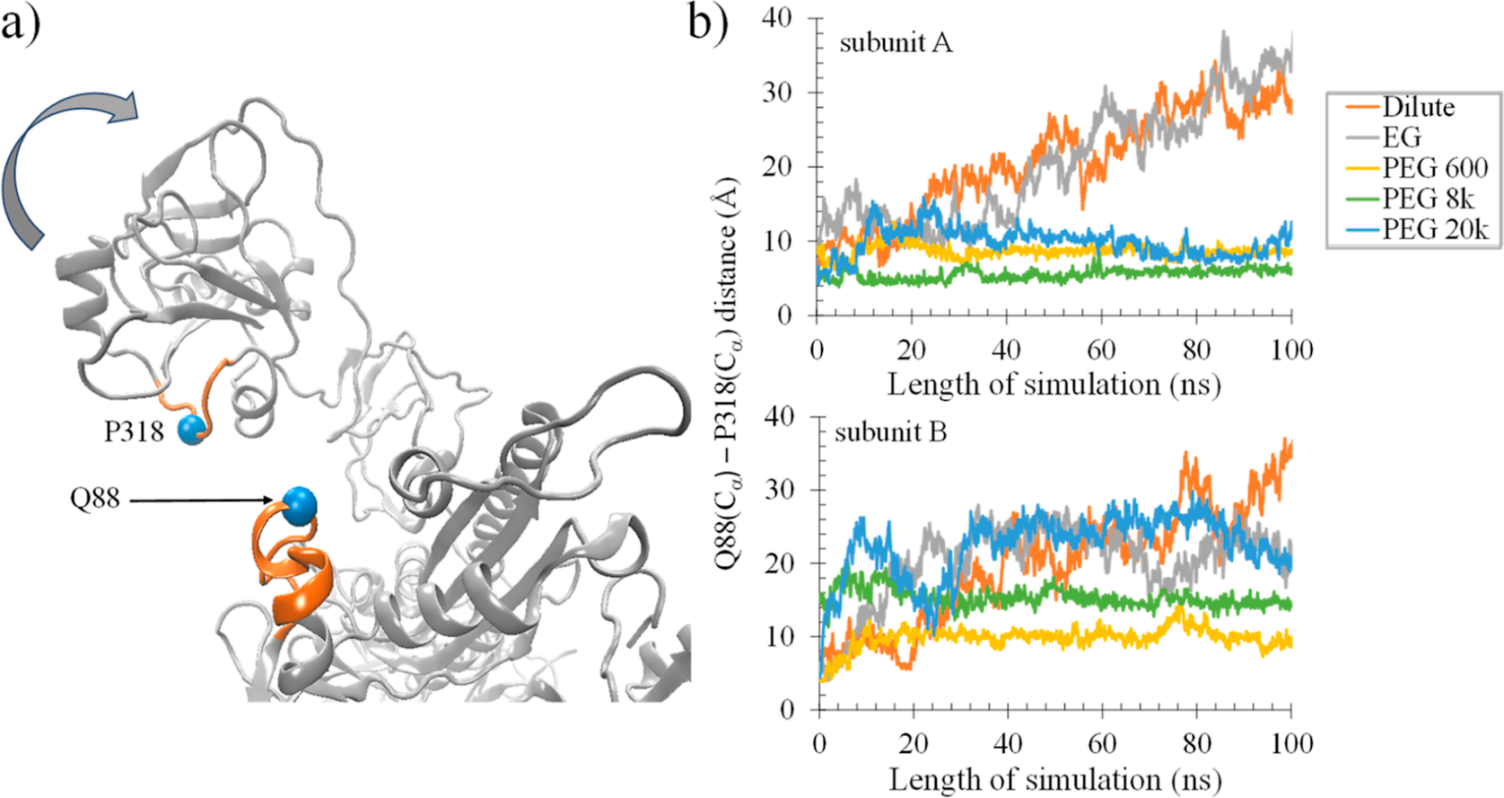
Dynamic changes in the active site cleft of Ec ProRS at the interface of editing (ED) and catalytic (CD) domains in the presence of various crowders. (a) ED and part of the CD that forms the active site cleft. The impact of crowders on the conformational change was assessed by monitoring the separation between the ED residues 313–322 and CD residues 84–93, which are indicated in orange. Residues P318 and Q88 are shown as blue spheres. (b) Plots showing the variation in the active site cleft distance for each subunit (between the C_α_ atom of Q88 and the C_α_ atom of P318) during the 100 ns MD simulation.

During the simulated dynamics, a conformational change occurs where the ED swings away from the catalytic domain (CD), creating a passage that facilitates the entry of substrates.^34^ The impact of crowders on this conformational change can be assessed by monitoring the separation between the ED residues 313–322 and CD residues 84–93. In the present analysis, the conformations were defined as “closed”, where the interdomain distance (i.e., Q88(C_α_)–P318(C_α_)) was ≤15 Å (Figure 8a), and “open”, if the interdomain distance was ^3^ > 15 Å.^34^ The cutoff of 15 Å was based on the active site conformation preventing the release of the bound U-shaped ATP.^33^ Analysis of the simulated trajectories revealed that the “open” conformational state is predominant (>75%) in the absence of crowder (Table 5, Figure 8b, orange line). The “open” state was also preferred in the presence of EG (Table 5, Figure 8b, gray line). The presence of polymer crowders resulted in a strong effect on the conformational ensemble. For PEG 600 and 8k, the ProRS was found to be exclusively in the “closed” conformational state (Table 5, Figure 8b). For the PEG 20k system, one of the subunits, i.e., subunit A (SUB A), was completely “closed” (Table 5, Figure 8b, top panel, blue line), while subunit B (SUB B) was in the “open” state (Table 5, Figure 8b, bottom panel, blue line). This difference originated because SUB A was in the proximity of PEG 20k, which wrapped its surface. On the other hand, both editing domains were found predominantly in the “open” conformation in the dilute condition (i.e., in the absence of crowders), demonstrating that PEG crowders drive the conformational equilibrium from an “open” to a “closed” state. This conformational shift is likely to impact protein function. Both *K*_M_ and *V*_max_ for Pro-AMP formation were impacted by PEG 8K crowders. The relative *K*_M_ (proline) decreased by ∼30% in the presence of 50 mg/mL PEG 8k, suggesting higher substrate affinity in the presence of crowder.^34^ Also, a ∼80% reduction in *V*_max_ is observed compared to the dilute condition. The “closed” conformation of the dimeric Ec ProRS in the presence of PEG crowders may be responsible for the observed decrease in *K*_M_ and *V*_max_, resulting in a significant reduction in the catalytic efficiency, i.e., *k*_cat_/*K*_M_. Crowder-induced conformational ensemble shifts in bacterial ProRS were previously observed for smaller crowders like dextrose and sucrose.^34^

### Altered Local Motions Evidenced in Per-Residue RMSF

**Figure 9:**
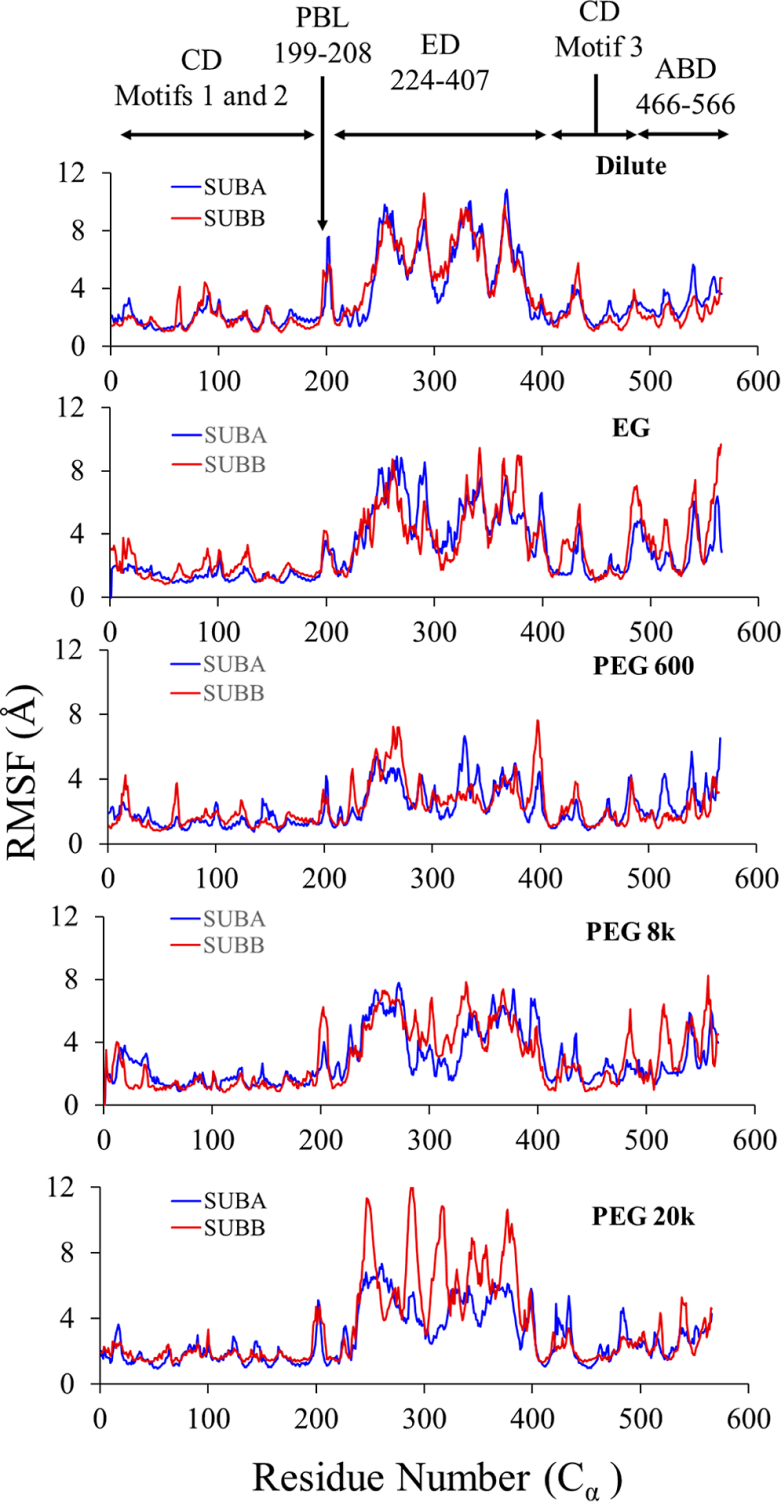
RMSF of alpha carbons (C_α_) of Ec ProRS in the absence and presence of PEG crowders of various MWs. The three domains, CD, ED, and ABD, represent the catalytic, editing, and anticodon binding domains, respectively, and PBL is the proline-binding loop.

The local fluctuations in various parts of the protein were examined by computing the RMSFs for the backbone C-alpha (C_α_) atoms for both subunits (SUB A and SUB B) of Ec ProRS using the 100 ns MD simulation data. Ec ProRS exhibited significant fluctuations in PBL and ED in the absence of crowders (Figure 9, top panel). Compared to the dilute condition, the dimeric protein exhibited similar backbone C_α_ atom flexibility in the presence of EG molecules. However, a substantial increase in the flexibility of the backbone was observed near the 260–270 region of the ED (Figure 9, second panel). A closer scrutiny of ED reveals a significant conformational change of a loop-helix-loop motif (residues 256 to 272 in Figure S3). This helix corresponds to the highly dynamic α2 helix of ProXp-ala, a free-standing ProRS ED homologue, and is known to be functionally relevant for substrate selection.^72^ The mobility of the loop-helix-loop motif was found to be reduced in PEG 600, PEG 8k, and one of the subunits of the ProRS dimer in contact with PEG 20k (Figure 9). The RMSF data showed a general trend of restricted backbone fluctuations with increased MW of the PEG (Figure 9, bottom three panels). Variations in the flexibilities of C_α_ atoms were noted between the two subunits, which could be due to the presence of different numbers of crowder molecules in the vicinity of SUB A and SUB B (Figure 9). For example, a significant difference in ED backbone fluctuations between the two subunits was noticed for the PEG 20k system. The higher fluctuation of ED C_α_ atoms of SUB B compared to SUB A is because the ED in SUB B was not encapsulated by PEG 20,000 polymers, whereas the ED of SUB A was wrapped by the polymeric chains. This observed difference in RMSFs for SUB A and SUB B confirmed that PEG 20k polymers impact protein flexibility through confinement. The impact of PEG crowders on the fluctuations of PBL and the ED domain dynamics may be responsible for the reduction in product (Pro-AMP) formation in the presence of crowders.^34^ The larger effect of PEG 600 compared to PEG 8k and 20k may also explain the greater impact on enzyme kinetics in the presence of PEG 600.^34^

### Evidence of Soft Interactions from Changes in the SASA

**Figure 10:**
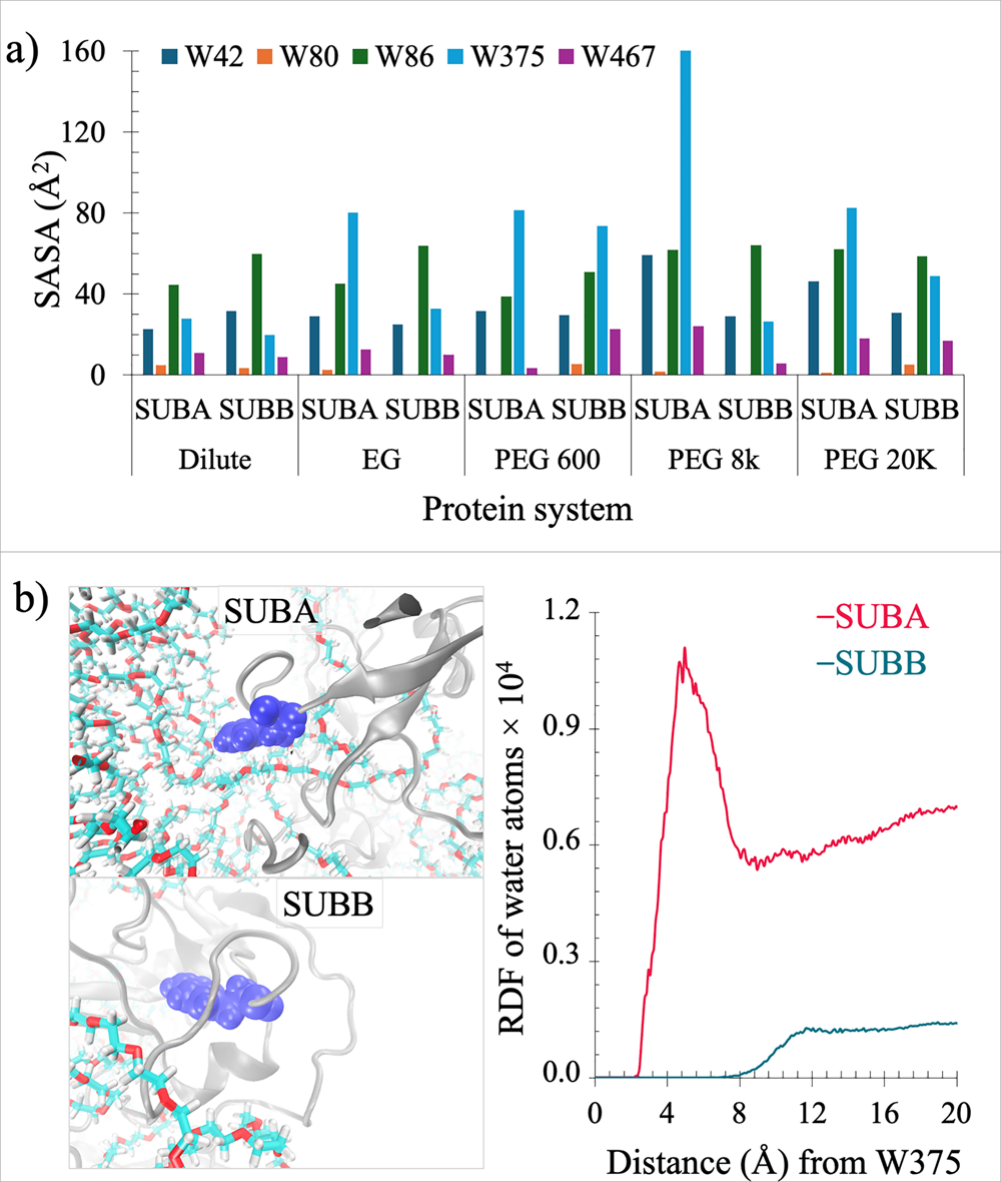
Effects of PEG crowders on soft interactions. (a) Computed SASA for the five tryptophans (W42, W80, W86, W375, and W467) in each subunit (SUB A and SUB B) of Ec ProRS in the dilute condition and in the presence of variable-sized PEG crowders. SASA was computed over 100 ns of the simulation. (b) Left: model showing W375 (blue space-filling representation) in the ED of SUB A and SUB B in the presence of PEG 8k. PEG 8k is in close proximity to SUB A and interacts noncovalently with it. Right: radial distribution function (RDF) of water molecules surrounding W375.

The MD simulation data were next analyzed to extract the SASA for the five tryptophan residues in each subunit of Ec ProRS (Figure 10). Conformational shifts due to the presence of crowder molecules that exhibit soft interactions with protein side chains are expected to affect the SASA of residues interacting with crowders. We expect increased SASA for Trp residues of Ec ProRS, whose surroundings are deprived of water due to the presence of a PEG molecule. Analysis of the SASA of individual Trp residues revealed a sharp increase in the SASA of W375 in the presence of crowders compared to that under the dilute condition. A slight increase in the SASA values for W42 and W86 was also observed. The changes in SASA in the presence of crowders varied between the two subunits. For W375, only SUB A exhibited increased SASA in the presence of PEG 8k and 20k, as this subunit is in close proximity to the polymer crowder (Figure 10b, left panel). To determine if the crowders impacted solvent (water) accessibility, a radial distribution function (RDF) between the water oxygen atom and the W375 endocyclic nitrogen atom (NE1) was computed using 100 conformations of 100 ns MD trajectories. The RDF plots (Figure 10b, right panel) demonstrate the probability of finding water or crowder molecules within the spherical region of W375; an increase in the RDF of water molecules surrounding W375 was observed. This difference in the SASA and RDF values for W375 between the two subunits indicates that the PEG molecules induce conformational changes through soft interactions with the protein side chains. These soft interactions are hydrophobic in nature and occur between the aliphatic protein side chains of SUB A and the methylene groups (−CH_2_−) of PEG crowders (Figure S4).

### PEG 20k Induces Protein Cluster Formation through Soft Interactions

**Figure 11:**
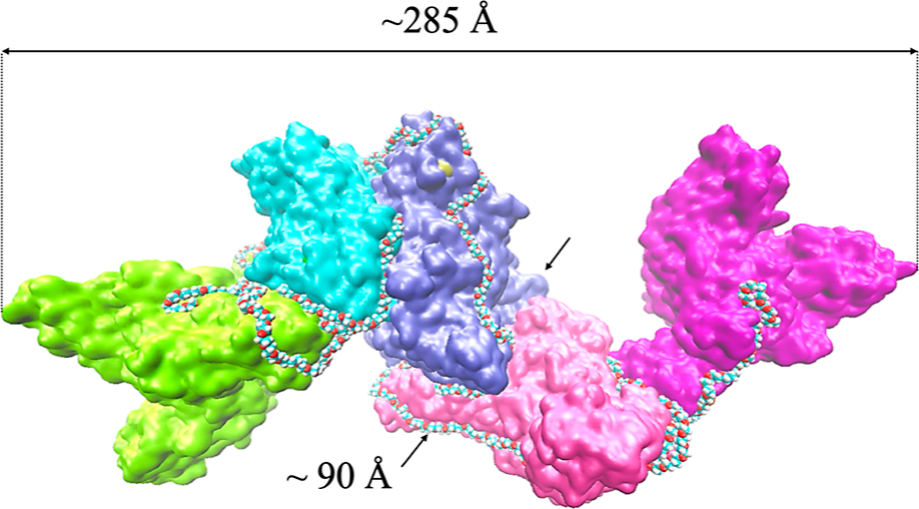
Aggregation of five Ec ProRS dimers on a PEG 20k chain as observed after a 100 ns MD simulation.

The MD simulations of Ec ProRS in the presence of PEG 20,000 demonstrated that the polymer wraps the protein (Figure 4d), which could cause the formation of aggregates. In addition, clustering of multiple dimeric ProRS on a PEG chain is possible. An assembly of five dimeric ProRS molecules wrapped by a PEG 20k molecule was allowed to evolve in water (Figure 11). The simulations revealed that the PEG aggregate wraps rapidly over the protein surface within the first nanosecond of the initiation of the simulations. The assembly remained intact throughout the 100 ns simulations, consistent with the observed soft interactions between the methylene groups (−CH_2_−) of PEG crowders and hydrophobic side chains of the protein molecules. The interactions contributed to the formation of a stable cluster comprising multiple Ec ProRS and PEG 20k.

## Conclusions

This study probed the molecular mechanism of the crowding effects of PEG molecules of variable sizes on the conformational dynamics of Ec ProRS. Our previous study, using end-point kinetics, showed a significant decrease in prolyl-adenylate formation in the presence of 100 mg/mL PEG 600.^34^ The present fluorescence study revealed that the smaller PEG does not significantly impact the conformation of ProRS, even at very high concentrations. Changes in both fluorescence intensity and maximum emission wavelength were modest in the presence of up to 400 mg/mL PEG 600, i.e., in the dilute regime (Table S1). However, the MD simulation data suggested that multiple PEG 600 polymers form clusters and impact the global dynamics and local flexibility of the ProRS backbone, as revealed by the RMSD and RMSF analyses. Hindered domain dynamics and increased rigidity of the dimeric structure were observed, as evident from the decreased radius of gyration of the Ec ProRS dimer. Furthermore, MD simulation results also demonstrated that the PEG600 clusters significantly reduced the global ED dynamics, altering the “open” to “closed” conformational equilibrium. In terms of impact on the local motional changes, a significant decrease in PBL and ED fluctuations were observed; dynamics in these regions was previously reported to be important for substrate binding and catalysis by Ec ProRS.^32^ Additionally, the simulations revealed that PEG 600 clusters physically block the entrance of the active site of Ec ProRS (Figure 5), which leads to a significant reduction in prolyl-adenylate formation in the presence of 100 mg/mL PEG 600.^34^

Large PEG molecules (8k and 20k) impacted protein conformation, as indicated by an increase in fluorescence quenching at higher crowder concentrations in dilute and semidilute solutions. AFM and melting experiments indicated the compactness of the ProRS structure in the presence of larger PEG crowders. This experimentally observed increased compactness was corroborated by MD simulations, which a demonstrated severe reduction in domain dynamics and flexibility. Thus, the larger PEG molecules create confinement as they wrap the dimeric protein via noncovalent interactions, as demonstrated by SASA analysis. As a result, PEG crowders reduce the global ED dynamics, altering the “open” to “closed” conformational equilibria. These alterations in the ED domain dynamics appeared to be responsible for the decreased catalytic efficiency of Ec ProRS.

Overall, the combined experimental and computational approaches enabled a molecular-level picture of the effects of PEG crowders on the modular Ec ProRS enzyme. The larger PEG molecules induced confinement, whereas the small PEGs caused physical crowding; both effects resulted in altered protein conformational dynamics and catalytic function. The present study reinforced that crowding effects are dependent on the crowder’s chemical nature, shape, and size, as well as on the target protein. This study contributes to our understanding of crowding and confinement effects in the cellular environment, with implications for the development of more potent and selective inhibitors for potential protein drug targets.

## Supporting Information Available

Overlap concentrations of various PEG molecules, impact of PEG molecular weight on intrinsic fluorescence of WT Ec ProRS, thermal stability measurement employing intrinsic fluorescence, conformational transition of an editing domain motif observed during MD simulations, and hydrophobic interactions between ProRS and PEG crowders (PDF)
The “closed” to “open” structural change (AVI)
Conformational change of the loop-helix-loop motif (AVI)

## Supplementary Material

bi3c00719_si_001.pdf

bi3c00719_si_002.avi

bi3c00719_si_003.avi

## Abbreviations

AFM: atomic force microscopy
BCM: Barycentric mean wavelength, λ
Ec: ProRS *Escherichia coli* prolyl-tRNA synthetase
EG: ethylene glycol
HOPG: highly-oriented pyrolytic graphite
MD: molecular dynamics
PEG: polyethylene glycol
WT: wild-type
