# Supplementary material for: Polyethylene Glycol Impacts Conformation and Dynamics of Escherichia coli Prolyl-tRNA Synthetase Via Crowding and Confinement Effects
Source: Biochemistry. 2024 Apr 12;63(13):1621–35. doi: 10.1021/acs.biochem.3c00719 (PMC11223479; doi:10.1021/acs.biochem.3c00719)
Supplement: Supplementary file 1 — bi3c00719_si_001.pdf [file bi3c00719_si_001.pdf]

SUPPLEMENTARY MATERIALS

Polyethylene Glycol Impacts Conformation and Dynamics of *Escherichia coli* Prolyl-tRNA Synthetase via Crowding and Confinement Effects

Jessica Liebau[$,1], Bethany Laatsch[$,1], Joshua Rusnak[1], Keegan Gunderson[1], Brianna Finke[1], Kassandra Bargender[1], Alex Narkiewicz-Jodko[1], Katelyn Weeks[1], Murphi T. Williams[1], Irina Shulgina[2], Karin Musier-Forsyth[*,2], Sudeep Bhattacharyya[*,1], and Sanchita Hati[*,1]

[1]Department of Chemistry & Biochemistry, University of Wisconsin-Eau Claire, Eau Claire, WI, 54702, USA.

[2]Department of Chemistry and Biochemistry and Center for RNA Biology, The Ohio State University, Columbus, OH, 43210, USA.

[$]These two authors have contributed equally to this work.

[*]To whom correspondence should be addressed: K.M.-F.: phone, (614) 292-2021; fax, (614)688-5402; e-mail, musier@chemistry.ohio-state.edu. S.B.: phone, (715) 836-2278; fax, (715) 836-4979; e-mail, bhattas@uwec.edu. S.H. phone, (715) 836-3850; fax, (715) 836-4979; e-mail, hatis@uwec.edu.

Table S1. The overlap concentration of PEG crowders that were used in the present study. The overlap concentrations were determined using the information provided in Ref. 1.[a]

PEG x 1000 (g/mol)	Overlap Concentration (% concentration)	Overlap Concentration (mg/mL)
**0.2**	1.1×10^2	1.1×10^3
**0.4**	70.	0.70×10^3
**0.6**	54	0.54×10^3
**1.0**	38	0.38×10^3
**2.0**	24	0.24×10^3
**4.0**	15	0.15×10^3
**8.0**	9.7	0.097×10^3
**20.0**	5.3	0.053×10^3

[a]The overlap concentration of an aqueous solution of a polymer is the critical concentration at which the polymer chains start to overlap with each other and form mesh-like networks, and as a consequence, interfere with any measurements involving concentration or size variations.

Reference:

1. Natalia Ziębacz, N., Wieczorek, S. A., Kalwarczyk, T., Fiałkowskia, M., and Hołyst, R. Crossover regime for the diffusion of nanoparticles in polyethylene glycol solutions: influence of the depletion layer. *Soft Matter* **2011**, ***7***, 7181-7186.

Figure S1. Impact of PEG molecular weight on intrinsic fluorescence of WT Ec ProRS. Sizes vary from 62.07 g/mol (EG) to a MW of 20,000 g/mol (PEG 20k). The sample "C" contains all PEG sizes and was prepared using equal volumes of each PEG solution. a) The relative fluorescence was calculated by the ratio of fluorescence intensity in the presence of PEG crowders to the fluorescence intensity of protein alone, which was set to 1.0. b) The barycentric mean wavelength in the presence of 50 mg/mL of crowders. The results presented here are an average of three trials with the mean (horizontal red lines) and standard deviation (vertical black lines) indicated.

a)

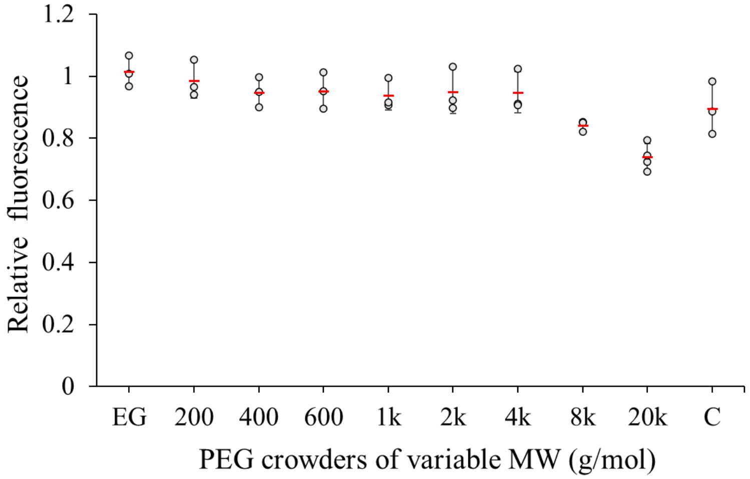

b)

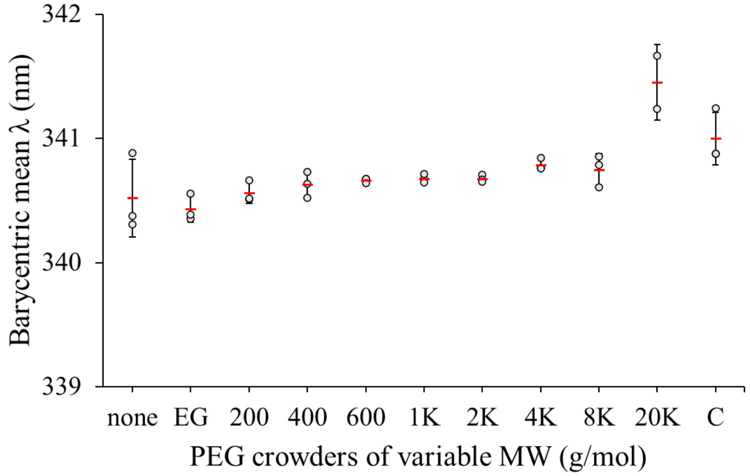

Figure S2. The thermal stability of Ec ProRS was studied by intrinsic fluorescence measurements. A representative plot is provided here for WT Ec ProRS in dilute conditions.

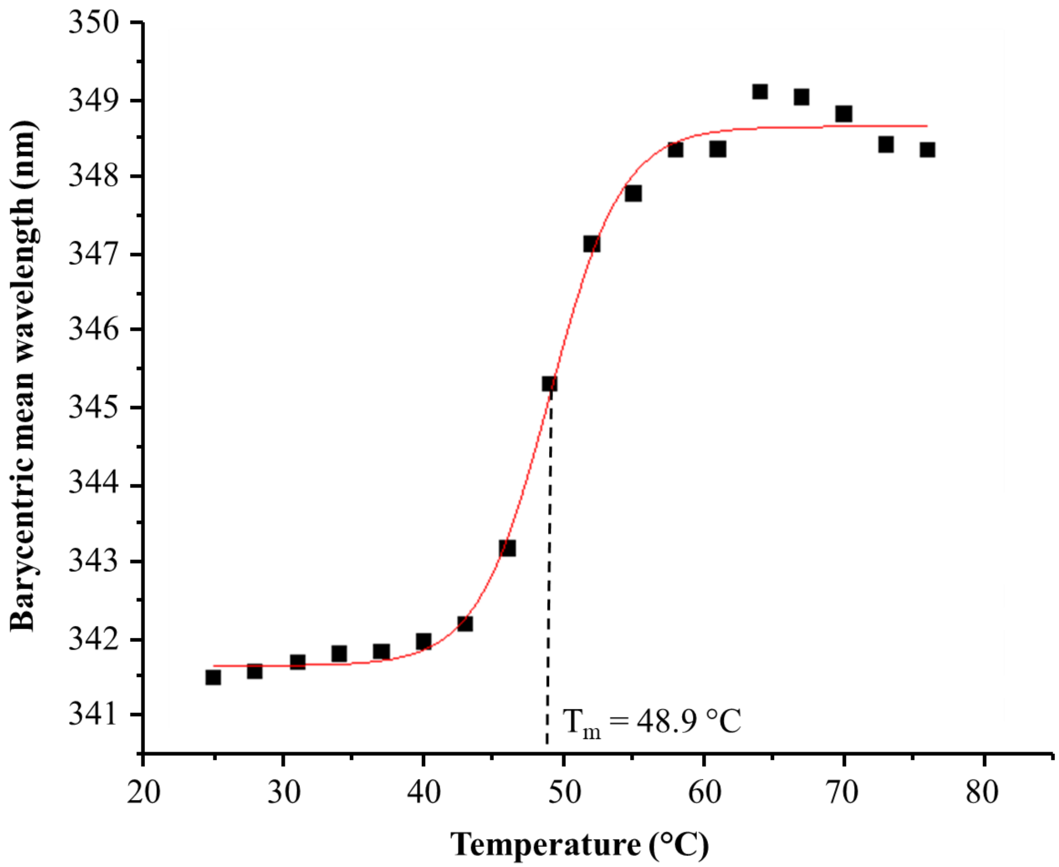

Figure S3. A notable conformational change of the loop-helix-loop motif (residues 252 to 267, shown in green) in the editing domain of Ec ProRS, was observed predominantly in the dilute and ethylene glycol-containing systems.

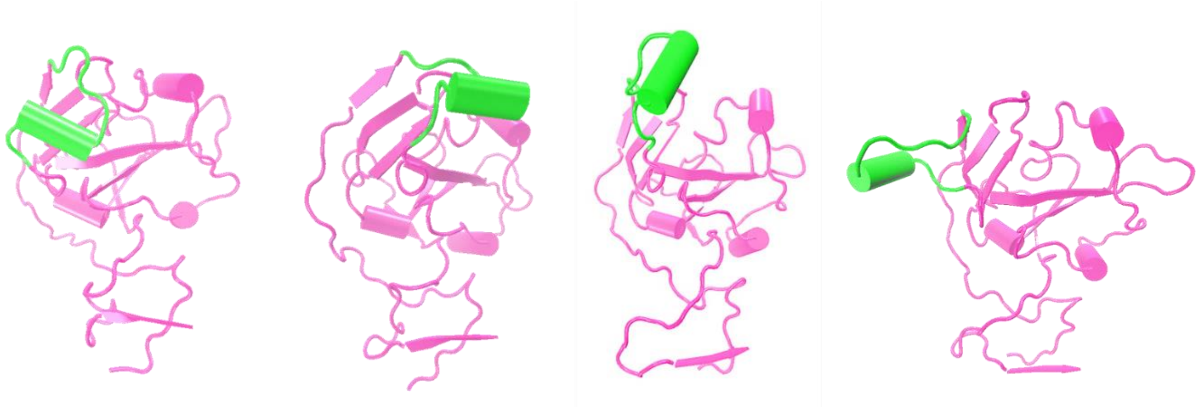

Figure S4. Hydrophobic interactions between aliphatic protein side chains and methylene groups (−CH_2−) of PEG crowders. The distances between the interacting carbon atoms were plotted along the simulated time of 100 ns. The three residues shown here are those surrounding W375 of SUB A of the Ec ProRS dimer (Fig. 11). This plot provides definitive evidence of soft PEG-protein interactions.

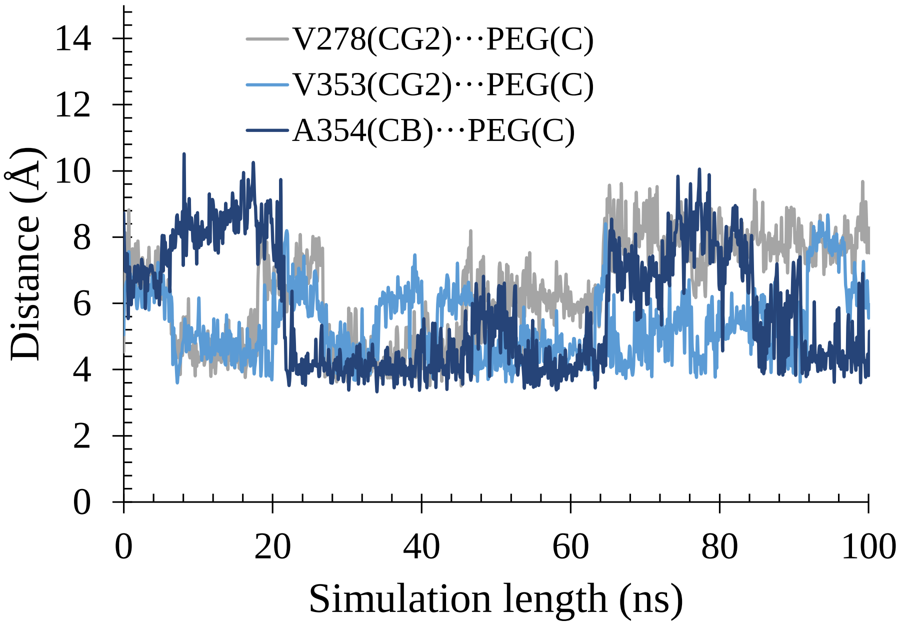

Movie clips

File name: Closed_to_open_480.avi

The "closed" to "open" structural change (shown in Fig. 7) observed in the 100 ns MD simulations of the dimeric Ec ProRS structure in dilute condition. The two editing domains, shown in pink, move further from each other during the simulation.

File name: LHL_conf_change_480.avi

The conformational change of the loop-helix-loop motif (residues 252 to 267, shown in green) in the editing domain of Ec ProRS observed in ethylene glycol (as shown in Fig. S3).
